# Association of Inflammatory–Hematological Biomarkers with Hypertension and Related Comorbidities

**DOI:** 10.3390/jcm15062279

**Published:** 2026-03-17

**Authors:** Evelina Maria Gosav, Daniela Maria Tanase, Anca Ouatu, Cristina Gena Dascalu, Oana Nicoleta Buliga-Finis, Diana Popescu, Andreea-Iustina Enache, Nicoleta Dima, Minerva Codruta Badescu, Ciprian Rezus

**Affiliations:** 1 Department of Internal Medicine, “Grigore T. Popa” University of Medicine and Pharmacy, 700115 Iasi, Romania; evelina.maria.gosav@umfiasi.ro (E.M.G.); anca.ouatu@umfiasi.ro (A.O.); oana-nicoleta.buliga-finis@umfiasi.ro (O.N.B.-F.); popescu.diana@umfiasi.ro (D.P.); andreea-iustina.enache@d.umfiasi.ro (A.-I.E.); nicoleta.dima@umfiasi.ro (N.D.); minerva.badescu@umfiasi.ro (M.C.B.); ciprian.rezus@umfiasi.ro (C.R.); 2 Internal Medicine Clinic, “St. Spiridon” County Clinical Emergency Hospital, 700111 Iasi, Romania; 3 Department of Medical Informatics and Biostatistics, Faculty of Dental Medicine, Grigore T. Popa University of Medicine and Pharmacy, 700115 Iasi, Romania

**Keywords:** high blood pressure, hypertension, HTN, neutrophil-to-lymphocyte ratio, NLR, platelet-to-lymphocyte ratio, PLR, chronic kidney disease, CKD, type 2 diabetes mellitus, T2DM

## Abstract

**Background:** According to current data, arterial hypertension (HTN) remains the leading cause of cardiovascular disease worldwide. Oftentimes, HTN is accompanied by type 2 diabetes mellitus (T2DM) and chronic kidney disease (CKD), interconnected by a pro-inflammatory pattern. Our study aimed to evaluate the roles of hematological serum cells, such as neutrophils, lymphocytes, and platelets, as well as the neutrophil-to-lymphocyte ratio (NLR) and platelet-to-lymphocyte ratio (PLR), in subjects with HTN, CKD, and/or T2DM. **Methods:** This retrospective unicentric study included 6077 patients admitted between 2018 and 2023; after applying exclusion criteria, patients were divided into groups for a comparative multivariate analysis. **Results:** The Mann–Whitney U test and the Kruskal–Wallis test showed statistically significant differences between groups. Higher neutrophil counts, lower lymphocyte counts, and platelet fluctuations were positively associated with HTN + comorbidities (p < 0.001 **). Receiver operating characteristic (ROC) analysis identified statistically significant associations for neutrophils and NLR in HTN (AUC = 0.442, p < 0.001 **, cut-off = 1.1217), for lymphocytes in HTN + T2DM (AUC sensitivity of 64.1%, cut-off = 18.950), for NLR (AUC 0.567, sensitivity of 50.6% and a specificity of 61.9% cut-off = 4.4174) and neutrophils (cut-off = 73.550) in HTN + CKD, and for NLR and PLR in HTN + CKD + T2DM (both having reliable AUC; sensitivity 87.9% and 81.8% for cut-off = 2.6957 and 10.5194, respectively). In all groups, AUC specificities were below the acceptable threshold (which considerably diminishes the practical clinical usability potential of these markers). **Conclusions:** This study demonstrated an association between hematological pro-inflammatory markers and hypertension and its comorbidities.

## 1. Introduction

Arterial hypertension (HTN) remains the leading cause of death and disability worldwide. Systolic blood pressure (SBP) tends to increase with age until the end of life, while diastolic blood pressure (DBP) levels increase until the 5th decade of life and decline after the 6th decade of life [1,2]. Given the local and systemic involvement of hematologic cells (neutrophils, lymphocytes, and platelets) in HTN [3], researchers have investigated their function and potential utility as biomarkers in CVD [4,5]. The neutrophil-to-lymphocyte ratio (NLR) and platelet-to-lymphocyte ratio (PLR) have attracted attention as easily obtained, cost-effective, inflammatory serological markers in various CVDs [6,7], comorbidities such as CKD [8], and T2DM, as well as their vascular complications [9].

As of now, the healthy range for NLR is reported to be around 1–3, with a mean of ~1.65, while most studies indicate that an optimal PLR in healthy adults ranges from 90 to 180. Higher levels are reported depending on the disease [10]. Although multiple studies have indicated an association between these markers and these pathologies, established cut-off values show inconsistent results across studies, suggesting differences in patient populations, statistical methods, and study designs [8,11,12,13]. For example, some authors reported that an NLR with a cut-off > 2.7 appears to be a good indicator of blood pressure variability in normotensive individuals [14]. Others have shown that both NLR and PLR biomarkers may help differentiate non-dipper from dipper HTN patients (NLR: 2.3 ± 0.9 versus 1.8 ± 0.5, *p < 0.001*, PLR: 117.7 ± 35.2 versus 100.9 ± 30.5, *p = 0.001*, respectively) [15]. In HTN with CKD [16] and HTN with T2DM [17], NLR often exceeds cut-offs of 2.2 or 3. PLR is reportedly higher in diabetes (typically 124–155) [18,19] and in CKD (though it may show less pronounced changes than NLR in specific CKD stages) [20,21]. We could not identify studies that investigated PLR in HTN with T2DM or CKD, or any research that combined the two biomarkers across all three disease associations.

Therefore, our original study aimed first to determine whether NLR and PLR values are associated with hypertension, diabetes, and renal disease. Additionally, we found it appropriate to investigate variations in neutrophil, lymphocyte, and platelet values to better understand the pathophysiological changes in these cases. Secondly, by establishing cut-off values for each parameter through comprehensive comparative analysis across groups, we can address knowledge gaps in these values and assess their clinical applicability.

## 2. Materials and Methods

### 2.1. Study Design and Extraction Data

This retrospective, unicentric cohort study included patients admitted to the Third Medical Clinic of Saint Spiridon Emergency Hospital in Iasi, Romania, over a period of 5 years (December 2018–December 2023). Among the 6077 patients, those who met the following criteria were evaluated: patients aged 18 to 75 years diagnosed with HTN, CKD, or T2DM. Firstly, patients over 75 years old were excluded, due to NLR and PLR heterogeneity, as their values rise independently with age [11], followed by AKI patients. Secondly, we also excluded primary factors for acute or chronic inflammation (which might affect the WBC and differential counts): active smoking, infections (like sepsis), and hematologic–oncological diseases (leukemia and cancer), arrhythmias (such as atrial fibrillation or atrial flutter, or ventricular arrhythmia), stroke, cardiomyopathy and coronary artery disease, severe liver disease, autoimmune diseases (such as systemic lupus erythematosus or rheumatoid arthritis), and patients who were on steroid medication or pregnant (n = 0).

**Figure 1:**
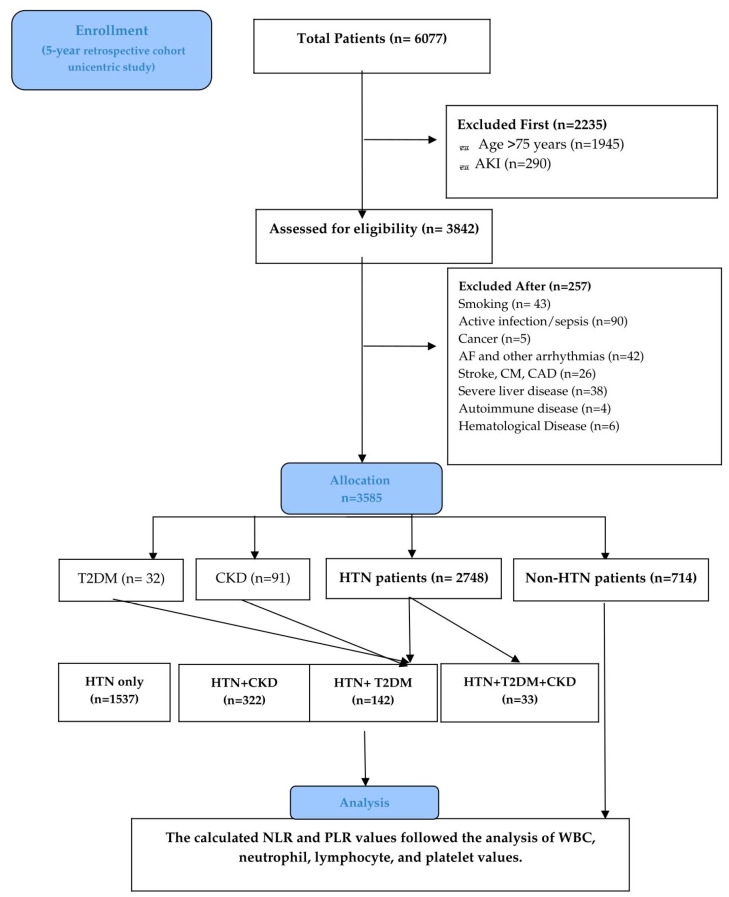
Consort flow diagram according to PRISMA guidelines for the included patients. Cardiomyopathy (CM); coronary artery disease (CAD); acute kidney injury (AKI); atrial fibrillation (AF); type 2 diabetes mellitus (T2DM); chronic kidney disease (CKD); white blood cell (WBC).

All diagnoses were obtained via ICD-10 codes and retrieved from InfoWorld, an electronic medical database that provides shared, comprehensive patient records across public hospitals and clinics. According to current guidelines, HTN was defined as blood pressure ≥ 130/80 mmHg, documented HTN, or receiving any antihypertensive therapy [1]. CKD diagnosis was asserted via the latest Kidney Disease: Improving Global Outcomes (KIDGO) guidelines definition: abnormalities of kidney structure or function present for a minimum of 3 months, with health implications, prior CKD diagnosis or newly found CKD based on GFR (mL/min per 1.73 m^2^), additional laboratory data (urinalysis and urine sediment), and imaging tests (abdominal ultrasound) [22]. Diabetes was established according to current antidiabetic treatment and documented disease or by the American Diabetes Association (ADA) guidelines, as determined by fasting plasma glucose (FPG) ≥ 126 mg/dL (≥7.0 mmol/L) on two separate occasions or a value for glycosylated hemoglobin (HbA1c) of 6.5% [23]. Comorbidities such as obesity, dyslipidemia, and anemia were also analyzed. The flowchart in Figure 1 shows the study design, process, and number of patients selected.

### 2.2. Laboratory Data and Ratio Calculation

Additional laboratory data were collected at admission, represented by complete blood count: lymphocyte, neutrophil, and platelet counts; hemoglobin level; renal function tests (creatinine, sodium, potassium, and urea); hepatic and lipid profiles; high-density lipoprotein cholesterol (HDL), low-density lipoprotein cholesterol (LDL), and total cholesterol; and triglyceride (TG), hemoglobin, and iron levels. For ratio calculations, we used the absolute counts of neutrophils, lymphocytes, and platelets from the complete blood count. NLR was calculated as absolute neutrophil count (cells/μL)/lymphocyte count (cells/μL), while PLR was also calculated using the formula absolute platelet count (cells/μL)/lymphocyte count (cells/μL).

### 2.3. Statistical Analysis

Statistical analysis was performed using SPSS 29.0 software. Frequencies, means, and standard deviations were used to describe variability. For multiple comparisons, post hoc tests following the Mann–Whitney U test and Kruskal–Wallis test were carried out, with pairwise comparisons using adjusted significance levels based on the Bonferroni correction (see supplementary tables and figures). The Kolmogorov–Smirnov test was used beforehand to assess the distribution of continuous variables and verify that they met the assumption of equal probabilities. Cut-off values for NLR and PLR, along with other parameters, were determined through receiver operating characteristic (ROC) analysis. An ROC curve with an AUC greater than 0.90 indicates excellent diagnostic performance; AUC values below 0.80, even if statistically significant, suggest limited clinical usefulness, while an AUC of 0.5 indicates no discrimination. A 95% confidence interval (CI) for the AUC that does not include 0.5 signifies statistical significance. Values of *p < 0.05* were considered statistically significant, and *p < 0.01* as highly significant.

### 2.4. Ethics Statement

This retrospective study was approved by the Institutional Review Board (IRB) and Ethics Committee of the Third Medical Clinic at Saint Spiridon Hospital in Iasi, as well as by the University of Medicine and Pharmacy “Grigore T. Popa” Iasi Research Ethics Committee. Patient consent was obtained at the time of hospital admission in accordance with hospital policies.

## 3. Results

This study investigated the role of blood cells in patients with hypertension and comorbidities. After applying the exclusion criteria, we initially assigned the remaining eligible patients (n = 3585) to the following groups: HTN patients (n = 2748), non-HTN patients (n = 714), T2DM-only patients (n = 32), and CKD-only patients (n = 91). Next, the HTN patients, along with their comorbidities, were divided into four groups—HTN-only patients (n = 1537), HTN + CKD patients (n = 322), HTN + T2DM patients (n = 142), and HTN + T2DM + CKD patients (n = 33)—for detailed multivariate analysis. The demographic characteristics of the patients, as well as the biological parameters, are shown in Table 1 and the Supplementary Materials.

### 3.1. Demographic Differences

HTN was the most frequently diagnosed condition; only 17.1% of cases had HTN combined with CKD or T2DM. Significant differences between genders were observed. An HTN diagnosis was more common in females than in males (58.0% versus 48.3%), and a similar pattern was noted for HTN with comorbidities; however, the differences between sexes were smaller in this case (Supplementary Table S1). These results may be due to differences in metabolic and hormonal profiles between genders. The age-related decline in estrogen levels increases the risk of CVDs in women. Recent studies describe estrogen’s dual role in regulating blood pressure [24]. When examining HTN incidence across different population groups, there appear to be no significant differences. HTN is slightly more common in urban areas than in rural areas. Interestingly, the proportion of HTN cases with CKD and T2DM is nearly identical across both environments. (Supplementary Table S2). These results may be explained by the fact that individuals in rural areas have lower access to medical offices/specialists and a lower financial status, while the majority remain undiagnosed, or are diagnosed later when other comorbidities and complications appear.

Further analysis revealed significant statistical differences between age groups (*p < 0.001*). As expected, most patients younger than 40 years had no disease (80.9%), while only 16.9% had HTN. With increasing age, the percentage of subjects without disease decreases. Subjects with HTN and T2DM or CKD have the highest percentage in those older than 60 years (Supplementary Table S3). In clinical practice, with age, the likelihood of encountering patients with multiple comorbidities increases. These results also align with recent global statistical data [1].

### 3.2. Presence of Anemia, Dyslipidemia, and Obesity

A comparative analysis stratified by anemia status also showed significant differences between groups (*p < 0.001*). Interestingly, patients with HTN and comorbidities were more likely to have anemia (24.8% vs. 16.1%) (Supplementary Table S4). Dyslipidemia appears to be statistically significantly associated with HTN and HTN+ comorbidities (*p < 0.001*). More than 50% percent of HTN patients had associated dyslipidemia, a higher percentage than in the HTN + CKD and HTN + T2DM groups (Supplementary Table S5). For obesity, a similar pattern is observed, with a stronger association with HTN (*p < 0.001*). Among obese patients, 60.1% have HTN, while 23.1% with obesity have HTN + CKD and/or T2DM (Supplementary Table S6). The results vary based on the number of patients in each group. They highlight that anemia, dyslipidemia, and obesity have a higher incidence in HTN, T2DM, and CKD patients. Anemia is a significant cardiovascular risk factor in HTN patients, creating a vicious cycle in which anemia fuels HTN and HTN worsens anemia via CKD [25]. As components of the metabolic syndrome, dyslipidemia, and obesity are associated with CVDs, CKD, and T2DM, and both influence blood pressure control through neurohormonal changes and arterial endothelial dysfunction [26]. Therefore, attention to these factors is important in clinical practice when evaluating a patient at risk.

### 3.3. Association of NLR in HTN Patients with T2DM and/or CKD 

Patients without disease had the lowest NLR values, with a slight increase in HTN patients and a significant increase in HTN patients with associated CKD (*p < 0.001*). Interestingly, the mean NLR was lower in patients with HTN and diabetes, but higher in subjects with HTN + CKD + T2DM (6.3148, ±6.35949). However, this difference is not statistically significant because the median values are relatively close. These results may be attributed, perhaps, to a slightly lower inflammatory profile in patients with HTN alone and diabetes (for example, antidiabetic drugs are known to exert significant anti-inflammatory and antioxidant effects beyond glycemic control. This aspect would be covered in the discussion section (Table 2, Supplementary Table S7, Figures S1 and S2).

### 3.4. Association of PLR in HTN Patients with T2DM and/or CKD

Additionally, using the Kruskal–Wallis test, PLR was found to vary significantly with HTN and HTN with comorbidities (*p* < 0.001). Similar to NLR, PLR varies in both directions: it is lower in patients with HTN or HTN + CKD + T2DM, and higher in those with HTN + CKD. The mean PLR in patients without HTN was 16.8774, with a median of 11.6150. These values decrease slightly, but not significantly, in individuals with HTN or HTN + T2DM. Conversely, PLR increases significantly in those with HTN + CKD and in the HTN + CKD + T2DM group. Although the difference is more noticeable here, statistical significance was not reached due to the small number of patients with the disease—only 33 cases compared to other groups. (Supplementary Tables S8 and S9, Figures S3 and S4). The HTN + CKD + T2DM group was the smallest, so the results should be interpreted with caution. A larger cohort is necessary for more accurate findings.

### 3.5. N, L, and P Variations in HTN and Comorbidities

For each parameter, the mean neutrophil count and median value dropped significantly in those with HTN alone, but only slightly in those with HTN and diabetes. Conversely, neutrophil levels rose significantly in patients with HTN and CKD (p < 0.001 **). A similar increase was observed in the last group. (Supplementary Tables S10 and S11, Figures S5 and S6). Not surprisingly, higher neutrophil levels are known to modulate oxidative stress and vascular response, can induce tissue inflammation and fibrosis, and promote endothelial damage [27].

The mean lymphocyte count and median were significantly higher in individuals with only HTN (mean 22.394 ± 9.8149, median 22.100) and in HTN with T2DM. In patients with HTN and CKD, however, lymphocyte counts were significantly lower. The same picture was seen in the HTN + CKD + T2DM group. Although the decrease was quite significant in the last category of patients, it did not reach statistical significance because the recorded median is very close to that in the first group (Supplementary Tables S12 and S13, Figures S7 and S8). Patients with diabetes, but especially those with CKD, often show significantly lower lymphocyte counts, which correlates with immune deficiency and higher infection risks [28].

Platelet fluctuations occur in both directions. In subjects with HTN, platelet levels are higher than in the non-HTN group. No other statistically significant fluctuations were observed, although it is notable that platelet levels continued to rise in patients with HTN accompanied by T2DM or CKD. Conversely, in patients with all three diseases, the mean platelet count was lower than in other groups. (Supplementary Tables S14 and S15, Figures S9 and S10). In diabetes and renal disease, platelets are characteristically hyperactive and increased in number; lower levels are seen when the two diseases are concomitant, particularly in advanced renal stages, and especially when microvascular complications are added due to chronic hyperglycemia (higher turnover) [29]. These results reflect the importance of the thrombotic status in these patients.

### 3.6. ROC Analysis in HTN and Comorbidities

Neither of the two ratios nor the parameters described were associated with essential hypertension. PLR showed no positive association. The AUC for lymphocytes and platelets was above 0.5, specificity was below 50%, and the AUC was <0.8, indicating very limited clinical usability of the test. Statistically significant results were also reported for NLR and neutrophil levels. These markers had very good sensitivity (97.3% and 97.1%), but their specificity was almost zero, which considerably diminishes their practical clinical usability (Table 3, Figures S11 and S12).

In the case of HTN accompanied by diabetes, the results are similar. Lymphocytes and NLR showed good sensitivity; unfortunately, their specificity was below the acceptable threshold of 50% (Supplementary Table S16, Figures S13 and S14).

**Figure 2:**
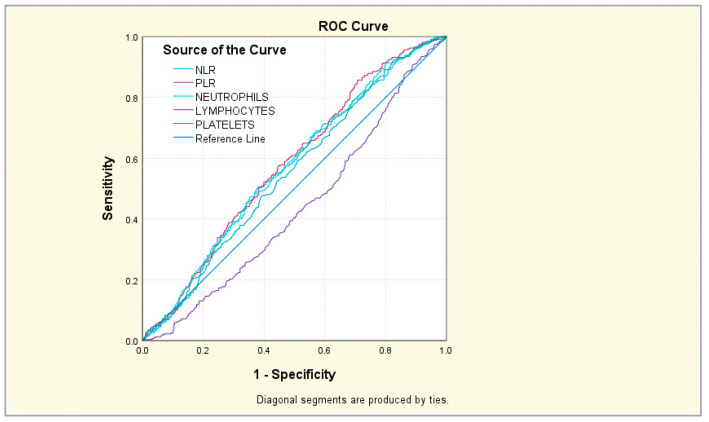
ROC curve of NLR, PLR, neutrophils, lymphocytes, and platelets in HTN + CKD.

The behavior of the five parameters is slightly improved when discussing an association with HTN + CKD. In this case, NLR showed good sensitivity and specificity at a cut-off value of 4.4174. The neutrophil level had relatively good specificity, although sensitivity was low. For the other three markers, although the AUC coefficients were relatively acceptable, the calculated cut-off values were notable only for their good sensitivity; the specificity, on the other hand, was well below the 50% acceptability threshold, which considerably diminishes their practical utility. All had AUCs above 0.5, close to 0.6, indicating poor discrimination (Supplementary Table S17 and Figure 2 and Figure S15).

Finally, in HTN with CKD and T2DM, again, no markers had good specificity. The NLR and PLR showed a good AUC; the proposed cut-off values had adequate sensitivities (87.9% and 81.8%), but specificity was low. The neutrophil level showed similar results; the lymphocyte level had almost zero specificity, and for the platelet level, both sensitivity and specificity were close to the 50% threshold, which also undermines the practical clinical utility of this indicator (Supplementary Table S18, Figures S16 and S17).

Overall, these results indicate an association between NLR, PLR, and other individual markers and HTN diagnosis and comorbidities. Although we achieved a cut-off for each individual parameter for each disease association, most biomarkers showed low specificity, often below clinically acceptable thresholds, which substantially limits their practical clinical utility. Also, the small group sizes of the last two groups, particularly HTN + T2DM + CKD, substantially limit the reliability of subgroup analyses.

## 4. Discussion

As proposed by Dr. Irvine Page in the 1940s, the first mosaic theory of hypertension and its later revision state that various interdependent renal, vascular, neural, and endocrine mechanisms contribute to the development of hypertension. In the last three decades, inflammation, oxidative stress, and microbiome have gained attention as contributors to the HTN mosaic. Among other roles, the vasculature participates in crosstalk between the endothelium and immune cells. Endothelial dysfunction in HTN is accompanied by monocyte activation, which stimulates proinflammatory cell production (cytokine and T-cell activation), alters peripheral blood leucocyte composition, and enhances platelet-dependent leucocyte adhesion and the risk of thrombosis [2]. Low-grade inflammation is a key driver of HTN development and hypertensive damage to target organs; therefore, hematological inflammatory cells have emerged as new potential biomarkers.

Regarding our investigated biomarkers, in hypertension, data show that NLR is involved in two immune pathways related to angiotensin II-mediated hypertensive and oxidative stress responses [12]. Over a 6-year follow-up involving 28,850 initially hypertension-free subjects, the authors demonstrated that higher NLR levels were linked to an increased risk of developing hypertension (*p* for trend *< 0.01*) [12]. NLR (cut-off > 2.7) appears to be a reliable indicator of blood pressure variability in normotensive individuals [14]. Conversely, a systematic review and meta-analysis found no difference in PLR values between patients with preeclampsia and those with normal pregnancies [30]. Both biomarkers may help differentiate non-dipper hypertensive patients from dipper hypertension. Significantly higher levels of PLR and NLR were registered in the non-dipper subjects (2.3 ± 0.9 versus 1.8 ± 0.5, *p < 0.001*, and 117.7 ± 35.2 versus 100.9 ± 30.5, *p = 0.001*, respectively) [15]. NLR and PLR are beneficial as inflammation detectors not only in primary HTN but also in secondary HTN (e.g., pulmonary HTN) [31] and other non-cardiac comorbidities and their associated vascular complications [9]. Interestingly, using stage-wise correlation and ROC curve analysis, Aneez et al. [8] noted that NLR and PLR were positively associated with proteinuria estimated by the protein-to-creatinine ratio in stages IIIA, IIIB, and IV of CKD, with predictive sensitivities and specificities exceeding 80%. The National Health and Nutrition Examination Survey (NHANES) registry showed the usefulness of NLR, PLR, and SII in assessing all-cause mortality among CKD patients [32]. These results indicate their utility in the progression of renal disease. Ongoing clinical trials are investigating NLR and/or PLR in HTN (preeclampsia–eclampsia, NCT01856387), CKD (NCT06670599), and T2DM (NCT06073756); these findings may offer new insights into their clinical utility.

Firstly, our study demonstrated significant demographic differences between groups, which align with current data. Interestingly, it has already been shown that peripheral blood inflammatory parameters—including neutrophil, lymphocyte, and platelet counts, NLR, PLR, the systemic immune inflammation index (SII), and the lymphocyte–monocyte ratio (LMR)—are associated with an increased risk of early menopause [33]. This 9-year cohort study, involving 6278 participants, showed that older men aged 60 years and older had a higher risk of incident HTN *(HR = 1.84*, *95% CI 1.06–3.18)* [7]. Additional comparative studies examining gender and age differences in relation to these biomarkers could be beneficial. Secondly, laboratory comorbidities such as anemia warrant further investigation and treatment in all patients [25]. Frequently seen in metabolic syndrome, obesity and dyslipidemia are major interconnected risk factors that also need proper screening and management [26]. Recently, experts defined the cardio–kidney–metabolic syndrome as a complex new entity that requires a multidisciplinary approach and specific guidelines [34].

Thirdly, our investigation demonstrated a relationship between these parameters and each disease; however, based on the AUC values determined, they showed limited discriminative power and thus no clinical applicability. These findings are consistent with most studies and suggest that NLR is linked to HTN development, with higher cut-off values associated with comorbidities. For example, a recent meta-analysis involving over seventy thosand patients indicated that NLR is a reliable predictor of HTN risk (OR = 1.11, 95% CI = 1.05–1.17, *p = 0.004*) and is associated with CVD and all-cause mortality [35]. Petramela et al. [36] found that in individuals with essential hypertension, NLR correlates with elevated blood pressure, a higher prevalence of dyslipidemia, obstructive sleep apnea syndrome, and subclinical organ damage. A retrospective observational study showed that hematological inflammatory markers, including NLR (*p < 0.001*), are useful for identifying non-dipper hypertensive patients [37]. The NHANES study demonstrated that NLR is a predictor of CVD in patients with diabetes [38]. Additionally, NLR is related not only to poor glycemic control and vascular complications in diabetes but also to declining glomerular filtration rates, proteinuria, and mortality in renal disease [39]. In both CKD and T2DM, NLR exceeds the healthy range, with higher levels in the presence of complications. For instance, Mertoglu et al. reported different cut-off values depending on diabetes status: NLR was significantly higher in prediabetic (1.60 ± 0.85), newly diagnosed diabetic (1.58 ± 0.78), and diabetic (2.07 ± 0.95) groups versus the no-disease group (1.37 ± 0.69) [13]. In CKD, NLR typically increases with disease severity, with reported values between 2.5 and 3.5 or higher, providing additional diagnostic value beyond eGFR and albuminuria [40]. In HTN + CKD and HTN + T2DM, depending on the context and population studied, NLR often exceeds cut-offs such as 2.2 or 3 [16,17]. To our knowledge, no study to date has established NLR cut-off values in patients with all three conditions.

Our study found that PLR levels vary in both directions and differ significantly between groups. Most of the literature suggests that higher PLR levels are found in non-dipper individuals. The NHANES registry provides additional information, showing a U-shaped relationship between PLR and all-cause mortality [24]. The authors reported that the median of PLR was significantly higher in the non-dipper group than in the dipper group [41]. Similar results were reported by Pinho et al. [42], indicating PLR’s potential as an indirect predictor of cardiovascular risk in primary hypertension (odds ratio [OR] = 2.11; 95% CI, 1.220–3.664; *p = 0.007*). Previously, Sunbul and colleagues [15] demonstrated that PLR (>107), but not NLR, was an independent predictor of non-dipper status. PLR values are significantly lower in prediabetes (90.35 ± 44.34) and early-stage T2DM (86.38 ± 45.24), but they increase in later stages [13]. In diabetic microvascular complications, SII, NLR, and PLR were linked to the risk of peripheral neuropathy, with only SII and PLR associated with diabetic nephropathy and retinopathy [43]. Interestingly, PLR and SII are both strong markers of inflammation and kidney injury in diabetes, with PLR showing a stronger association with proteinuria (PLR = 252.09 versus 99.74, *p < 0.001*) and the urine albumin-to-creatinine ratio [44]. Because subclinical damage precedes significant albuminuria in CKD and diabetic nephropathy, urea, creatinine, and ACR have limited value for early detection. Therefore, a panel of easily measurable pro-inflammatory markers could help prevent disease onset and progression, ultimately improving patient outcomes.

When assessing individual peripheral blood components, consistent with our findings, others have reported that non-dipper patients have significantly lower mean white blood cell counts and lymphocyte counts (*p = 0.001*) [41]. Most relevant studies indicate that a higher WBC count can independently predict hypertension [45,46]. Through observational and genetic analyses, Siedlinski et al. [47] identified a potential causal relationship between lymphocyte count and systolic and diastolic BP. Additionally, elevated platelet levels are associated with increased diastolic blood pressure and, to a lesser extent, systolic blood pressure [48]. In T2DM, platelets are initially hyperactive, increasing the risk of thromboembolic events, whereas in CKD, they become more dysfunctional, leading to a balance between bleeding and thrombosis. In these cases, the variability in platelet values across groups is explainable, given that this status is characteristic of a specific disease association.

This study has some limitations. (i) Our data were collected retrospectively from ICD-10 codes; therefore, we could not determine the disease stages. It is also important to note the lack of ambulatory blood pressure data and the absence of adjustment for inflammatory confounders beyond the exclusion criteria. Similarly, the absence of disease staging (e.g., CKD stage, glycemic control, or treatment status at the time of admission) limits interpretability. (ii) The patients could not be questioned about their dipper or non-dipper status, hypertension severity, CKD stage, or T2DM complications, which would have improved the interpretation of the results. (iii) Given the limitations of obtaining medication or drug data from our electronic medical records at the time of admission, the effects of cardiovascular or diabetic therapies on hematological indices may influence the findings. For example, some classes of cardiovascular medications and antidiabetics have pleiotropic anti-inflammatory effects or influence platelet levels [49]. (iv) Because this is a unicentric study, the included patients may differ in socioeconomic status, environmental behaviors, and race from the general population; external validation should be performed to exclude selection bias.

## 5. Conclusions

Hypertension remains a global problem, along with metabolic disease and kidney disease, leading to high health costs and an increased risk of mortality. This study demonstrated that hematological parameters—neutrophils, lymphocytes, and platelets, NLR, and PLR—are positively associated with HTN and comorbidities. The different variations and cut-off values identified in this research provide high sensitivity but near-zero specificity, and based on the AUC values determined, they showed limited discriminative power making them unlikely to be clinically actionable. These parameters are disease-dependent, so larger studies are needed to establish definitive values and their clinical utility in complex pathologic associations.

## Figures and Tables

**Table 1:** Laboratory parameters in HTN patients.

	N	Mean	Std. Deviation	Min	Max	Median	IQR 25th–75th
Days in hospital	2069	5.59	2.788	0.00	30.00	5.00	4.00–7.00
NLR	2034	4.57	4.43	0.19	68.50	3.17	2.15–5.36
PLR	2034	15.45	13.45	0.24	173.68	11.36	8.01–17.61
Neutrophils × 10^3^ μL	2034	67.85	11.40	9.50	95.90	67.60	60.10–76.00
Lymphocytes × 10^3^ μL	2034	21.63	9.77	1.40	80.00	21.30	14.28–28.10
Platelet × 10^3^ μL	2034	249.55	85.33	11.00	821.00	242.00	196.00–291.00
Hb (g/dL)	1850	13.03	2.18	3.00	21.10	13.30	12.10–14.40
Leucocytes × 10^3^ μL	2019	12.09	59.63	0.00	2332.10	8.28	6.15–10.62
CRP (mg/dL)	1758	2.54	5.07	0.02	34.47	0.63	0.24–2.08
Fibrinogen (mg/dL)	1228	377.89	92.40	107.00	749.00	367.50	319.00–424.00
Na (mmol/L)	2054	138.77	5.60	98.00	149.00	140.00	138.00–142.00
K (mmol/L)	1955	4.31	0.58	1.80	7.60	4.30	4.00–4.60
Urea (mg/dL)	2058	44.62	23.02	8.00	296.00	40.00	31.00–51.00
Creatinine (mg/dL)	2058	0.96	0.44	0.33	9.95	0.85	0.73–1.05
HbA1c (%)	1179	6.79	1.58	4.30	14.90	6.30	5.70–7.30
CK (mg/dL)	1349	189.37	947.85	11.00	30,318.00	87.00	55.00–144.00
CK-MB (mg/dL)	1146	25.42	30.32	6.00	557.00	20.00	15.00–27.00
NT-proBNP (pg/dL)	271	2873.60	4652.08	5.18	29,697.00	1031.00	193.00–3372.00
hsTnI (ng/mL)	229	47.14	140.99	0.00	1209.00	9.18	4.20–24.95
Total cholesterol (mg/dL)	1933	175.97	53.48	44.00	493.00	169.00	138.00–208.50
LDL (mg/dL)	569	105.56	45.25	13.00	422.00	100.00	73.00–134.00
HDL (mg/dL)	1921	44.66	15.77	5.00	167.00	43.00	34.00–53.00
TG (mg/dL)	1912	131.39	95.16	18.00	1998.00	111.00	82.00–155.00
Uric acid (mg/dL)	1820	4.84	3.20	0.00	15.50	5.45	2.90–7.00
Ferritin (mg/dL)	1408	181.42	258.74	3.00	3955.00	108.00	59.00–206.00
Iron (mg/dL)	1523	69.17	37.98	6.00	350.00	63.00	41.00–89.00
AST (U/L)	1901	30.40	44.63	6.00	1360.00	22.00	17.00–29.00
ALT (U/L)	2018	31.64	48.72	4.00	879.00	22.00	16.00–33.00

NLR—neutrophil-to-lymphocyte ratio; PLR—platelet-to-lymphocyte ratio; CRP—C-reactive protein; Hb—hemoglobin; Na—sodium; K—potassium; CK—creatine kinase; CK-MB—creatine phosphokinase–MB; NT-proBNP—N-terminal prohormone of brain natriuretic peptide; hsTnI—high-sensitivity troponin I; TG—triglyceride; ALT—alanine transaminase; AST—aspartate transaminase; mean ± SD, the interquartile range (IQR).

**Table 2:** The Kruskal–Wallis H test-comparative analysis of the NLR in HTN, with/without CKD or T2DM.

NLR							IQR		Kruskal–Wallis H Test
	N	Mean	Std. Deviation	Min	Max	Median	25th	75th	
HTN + comorbidities									
absent	714	5.2540	5.04544	0.46	38.25	3.5564	2.2374	6.1567	H = 68.668
HTN	1537	4.3173	4.25327	0.21	68.50	2.9819	2.0625	4.9603	p < 0.001 **
TDM + HTN	142	4.4057	4.57371	0.78	40.39	3.0981	2.0623	5.2781	
CKD + HTN	322	5.6957	4.77211	0.19	33.29	4.4376	2.6348	6.9092	
TDM + CKD + HTN	33	6.3148	6.35949	2.06	35.54	3.9368	3.0160	7.3360	

T2DM—type 2 diabetes mellitus; CKD—chronic kidney disease; HTN—hypertension; N—number of patients; p < 0.001 **—high statistical significance.

**Table 3:** ROC analysis of NLR, PLR, neutrophils, lymphocytes, and platelets in HTN.

Diagnostic: HTN	AUC	*p-Value*	95% CI		Gini Index	Cut-Off Value	Sensitivity	Specificity
			L.inf	L.sup				
NLR	0.442	0.000 **	0.416	0.468	−0.116	**1.1217**	0.973	0.038
PLR	0.478	0.097	0.451	0.504	−0.045	**5.9158**	0.900	0.143
Neutrophils/μL	0.453	0.000 **	0.427	0.479	−0.094	**46.550**	0.971	0.045
Lymphocytes/μL	0.561	0.000 **	0.535	0.587	0.122	**18.850**	0.630	0.483
Platelets/μL	0.546	0.001 **	0.519	0.573	0.092	**219.50**	0.633	0.475

HTN—hypertension; p < 0.001 **—high statistical significance.

## Data Availability

The original contributions presented in this study are included in the article. Further inquiries can be directed to the corresponding authors.

## Supplementary Materials

The following supporting information can be downloaded at: https://www.mdpi.com/article/10.3390/jcm15062279/s1.
