# Supplementary material for: Association of Inflammatory–Hematological Biomarkers with Hypertension and Related Comorbidities
Source: J Clin Med. 2026 Mar 17;15(6):2279. doi: 10.3390/jcm15062279 (PMC13026883; doi:10.3390/jcm15062279)
Supplement: Supplementary file 1 [file jcm-15-02279-s001.zip › jcm-4098889-supplementary.pdf]

SUPPLEMENTARY

Table S1. Distribution of HTN patients associated with comorbidities according to gender.

**HTN+ Comorbidities**	**Gender**						**Pearson Chi-squared test**
	**Male**		**Female**		**Total**		
	N	%	N	%	N	%	
absent	421	30.3%	313	20.3%	734	25.0%	Chi2 = 50.708
T2DM	20	1.4%	12	0.8%	32	1.1%	*p <0.001***
CKD	49	3.5%	42	2.7%	91	3.1%	
T2DM + CKD	5	0.4%	3	0.2%	8	0.3%	
HTN	671	48.3%	896	58.0%	1567	53.4%	
T2DM + HTN	59	4.2%	84	5.4%	143	4.9%	
CKD + HTN	151	10.9%	175	11.3%	326	11.1%	
T2DM + CKD + HTN	13	0.9%	20	1.3%	33	1.1%	
**Total**	**1389**	**100.0%**	**1545**	**100.0%**	**2934**	**100.0%**	

T2DM—type 2 diabetes mellitus; CKD—chronic kidney disease; HTN-Hypertension; N-number of patients; p <0.001 **—high significative statistic.

Table S2. Distribution of patients by origin and environment.

**HTN+ Comorbidities**	**Environment**						**Pearson Chi-squared test**
	**Urban**		**Rural**		**Total**		
	N	%	N	%	N	%	
absent	296	22.9%	438	26.6%	734	25.0%	Chi2 = 8.363
T2DM	16	1.2%	16	1.0%	32	1.1%	*p = 0.302*
CKD	37	2.9%	54	3.3%	91	3.1%	
T2DM + CKD	2	0.2%	6	0.4%	8	0.3%	
HTN	719	55.7%	848	51.6%	1567	53.4%	
T2DM + HTN	63	4.9%	80	4.9%	143	4.9%	
CKD + HTN	143	11.1%	183	11.1%	326	11.1%	
T2DM + CKD + HTN	14	1.1%	19	1.2%	33	1.1%	
**Total**	**1290**	**100.0%**	**1644**	**100.0%**	**2934**	**100.0%**	

*T2DM—type 2 diabetes mellitus; CKD—chronic kidney disease; HTN-Hypertension; N-number of patients; p <0.001 **—high significative statistic.*

Table S3. Distribution of HTN, CKD, and/or TDM patients depending on age groups.

HTN+ Comorbidities	Age groups						Total		Pearson Chi-squared test
	< 40 years		40-60 years		> 60 years				
	N	%	N	%	N	%	N	%	
absent	72	80.9%	254	38.6%	408	18.7%	734	25.0%	Chi2 = 294.729
T2DM			7	1.1%	25	1.1%	32	1.1%	*p <0.001***
CKD			10	1.5%	81	3.7%	91	3.1%	
T2DM + CKD			2	0.3%	6	0.3%	8	0.3%	
HTN	15	16.9%	328	49.8%	1224	56.0%	1567	53.4%	
T2DM + HTN			29	4.4%	114	5.2%	143	4.9%	
CKD + HTN	2	2.2%	27	4.1%	297	13.6%	326	11.1%	
T2DM + CKD+ HTN			1	0.2%	32	1.5%	33	1.1%	
**Total**	**89**	**100.0%**	**658**	**100.0%**	**2187**	**100.0%**	**2934**	**100.0%**	

T2DM—type 2 diabetes mellitus; CKD—chronic kidney disease; HTN-Hypertension; N-number of patients; p <0.001 **—high significative statistic.

Table S4. Distribution of HTN, CKD, and/or TDM patients depending on the presence of anemia.

HTN+ Comorbidities	ANEMIA				Total		Pearson Chi-squared test
	absent		present				
	N	%	N	%	N	%	
absent	633	24.5%	101	28.5%	734	25.0%	Chi2 = 87.885
T2DM	27	1.0%	5	1.4%	32	1.1%	*p <0.001***
CKD	66	2.6%	25	7.0%	91	3.1%	
T2DM + CKD	4	0.2%	4	1.1%	8	0.3%	
HTN	1435	55.6%	132	37.2%	1567	53.4%	
T2DM + HTN	131	5.1%	12	3.4%	143	4.9%	
CKD + HTN	261	10.1%	65	18.3%	326	11.1%	
T2DM + CKD + HTN	22	0.9%	11	3.1%	33	1.1%	
**Total**	**2579**	**100.0%**	**355**	**100.0%**	**2934**	**100.0%**	

T2DM—type 2 diabetes mellitus; CKD—chronic kidney disease; HTN-Hypertension; N-number of patients; p <0.001 **—high significative statistic.

Table S5. Distribution of HTN, CKD, and/or TDM patients depending on the presence of dyslipidemia.

HTN+ Comorbidities	DYSLIPIDEMIA				Total		Pearson Chi-squared test
	absent		present				
	N	%	N	%	N	%	
absent	575	31.4%	159	14.4%	734	25.0%	Chi2 = 145.895
T2DM	23	1.3%	9	0.8%	32	1.1%	*p <0.001***
CKD	77	4.2%	14	1.3%	91	3.1%	
T2DM + CKD	5	0.3%	3	0.3%	8	0.3%	
HTN	867	47.4%	700	63.4%	1567	53.4%	
T2DM + HTN	72	3.9%	71	6.4%	143	4.9%	
CKD + HTN	188	10.3%	138	12.5%	326	11.1%	
T2DM + CKD + HTN	23	1.3%	10	0.9%	33	1.1%	
**Total**	**1830**	**100.0%**	**1104**	**100.0%**	**2934**	**100.0%**	

T2DM—type 2 diabetes mellitus; CKD—chronic kidney disease; HTN-Hypertension; N-number of patients; p <0.001 **—high significative statistic.

Table S6. Distribution of HTN, CKD, and/or TDM patients depending on the presence of obesity.

HTN+ Comorbidities	Obesity				Total		Pearson Chi-squared test
	absent		present				
	N	%	N	%	N	%	
absent	617	30.5%	117	12.9%	734	25.0%	Chi2 = 132.906
T2DM	20	1.0%	12	1.3%	32	1.1%	*p <0.001***
CKD	71	3.5%	20	2.2%	91	3.1%	
T2DM + CKD	5	0.2%	3	0.3%	8	0.3%	
HTN	1020	50.4%	547	60.1%	1567	53.4%	
T2DM + HTN	68	3.4%	75	8.2%	143	4.9%	
CKD + HTN	205	10.1%	121	13.3%	326	11.1%	
T2DM + CKD + HTN	18	0.9%	15	1.6%	33	1.1%	
**Total**	**2024**	**100.0%**	**910**	**100.0%**	**2934**	**100.0%**	

T2DM—type 2 diabetes mellitus; CKD—chronic kidney disease; HTN-Hypertension; N-number of patients; p <0.001 **—high significative statistic.

Table S7. Pairwise Comparisons analysis between diagnostics for NLR

**Pairwise Comparisons of HTN + comorbidities**	Test Statistic	*p-value*
HTN vs. T2DM+ HTN	-13.976	1.000
HTN vs. absent	161.167	0.000**
HTN vs. CKD+ HTN	-352.726	0.000**
HTN vs. T2DM +CKD+ HTN	-443.190	0.015*
T2DM + HTN vs. absent	147.191	0.435
T2DM +HTN vs. CKD+ HTN	-338.750	0.000**
T2DM +HTN vs. T2DM +CKD+ HTN	-429.214	0.051
absent vs. CKD+ HTN	-191.558	0.003**
absent vs. T2DM +CKD+ HTN	-282.023	0.459
CKD+ HTN vs. T2DM +CKD+ HTN	-90.464	1.000

T2DM—type 2 diabetes mellitus; CKD—chronic kidney disease; HTN-Hypertension; p <0.001 **—high significative statistic.

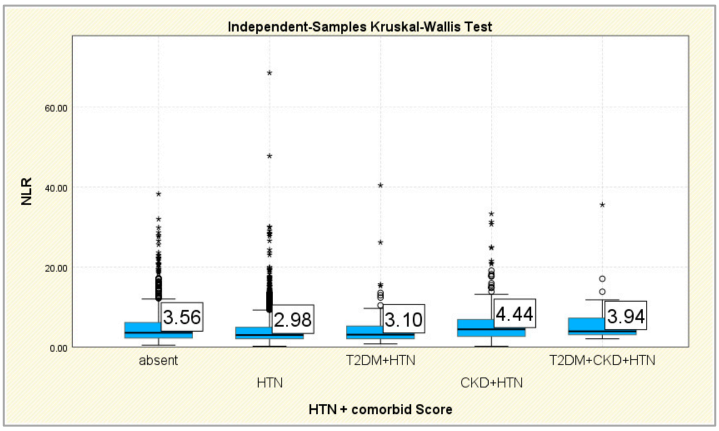

Figure S1. Independent-Sample Kruskal-Wallis Test analysis between diagnostics for NLR

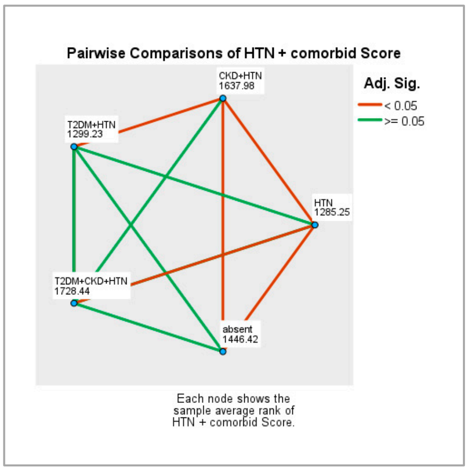

Figure S2. Pairwise Comparisons analysis between diagnostics for NLR

Table S8. The Kruskal–Wallis H test-comparative analysis of the PLR depends on the presence of HTN, with/without CKD or TDM

							IQR		**Kruskal-Wallis**
**PLT**	N	Mean	Std. Deviation	Min	Max	Median	25th	75th	**H test**
**HTN + comorbidities**									
absent	714	16.8774	16.68316	0.91	172.97	11.6150	7.5182	19.1964	**H = 42.714**
HTN	1537	14.6795	12.75329	0.24	173.68	10.9934	7.7630	16.4121	*p <0.001***
TDM+HTN	142	14.6697	11.56335	2.85	73.82	10.6173	7.7230	17.9729	

CKD+HTN	322	19.0358	15.86925	2.88	112.71	13.7778	9.4350	22.7587
TDM+CKD+HTN	33	19.5134	19.73975	5.98	115.38	13.2547	10.6787	21.8293

T2DM—type 2 diabetes mellitus; CKD—chronic kidney disease; HTN-Hypertension; N-number of patients; p <0.001 **—high significative statistic.

Table S9. Pairwise Comparisons analysis between diagnostics for PLR

**Pairwise Comparisons of HTN + comorbid**	Test Statistic	*p-value*
HTN vs. DZ+HTN	-0.856	1.000
HTN vs. absent	65.055	0.702
HTN vs. BRC+HTN	-300.132	0.000**
HTN vs. T2DM+CKD+HTN	-325.003	0.199
T2DM+HTN vs. absent	64.198	1.000
T2DM+HTN vs. CKD+HTN	-299.275	0.002**
T2DM+HTN vs. T2DM+CKD+HTN	-324.147	0.345
absent vs. CKD+HTN	-235.077	0.000**
absent vs. T2DM+CKD+HTN	-259.948	0.658
CKD+HTN vs. T2DM+CKD+HTN	-24.871	1.000

T2DM—type 2 diabetes mellitus; CKD—chronic kidney disease; HTN-Hypertension; p <0.001 **—high significative statistic.

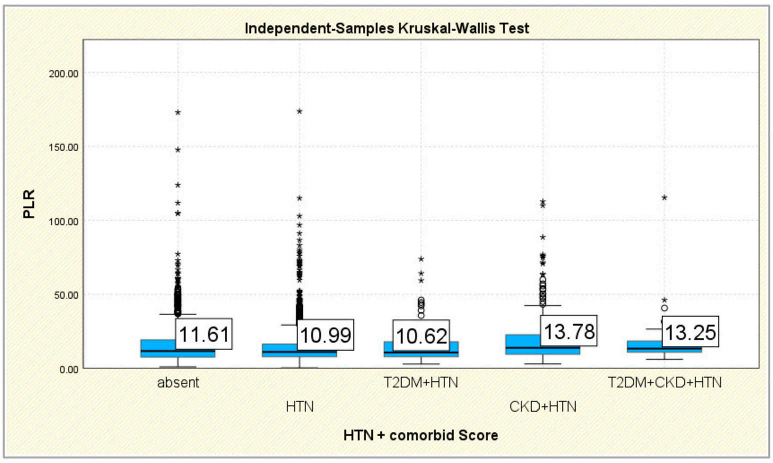

Figure S3. Independent-Sample Kruskal-Wallis Test analysis between diagnostics for PLR

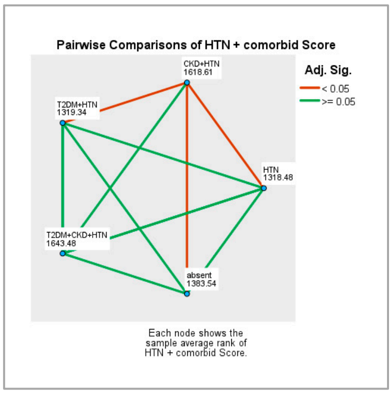

Figure S4. Pairwise Comparisons analysis between diagnostics for PLR

Table S10. The Kruskal–Wallis H test-comparative analysis of the absolute value of neutrophils on the presence of HTN, with/without CKD or TDM

Neutrophils%	N	Mean	Std. Deviation	Min	Max	Median	IQR 25th	IQR 75th	Kruskal-Wallis H test
**HTN + comorbidities**									
absent	714	68.751	12.0863	29.5	95.1	68.850	60.775	77.225	**H = 60.735**
HTN	1537	66.941	11.4190	9.5	95.9	66.500	59.100	75.100	*p <0.001***
T2DM+HTN	142	67.523	11.0137	38.1	93.0	67.350	59.575	74.775	
CKD+HTN	322	71.694	10.7515	15.3	95.1	71.750	63.750	79.700	
T2DM+CKD+HTN	33	73.839	8.6678	59.5	92.4	73.300	66.700	81.900	

T2DM—type 2 diabetes mellitus; CKD—chronic kidney disease; HTN-Hypertension; N-number of patients; p <0.001 **—high significative statistic.

Table S11. Pairwise Comparisons analysis between diagnostics for neutrophils

**Pairwise Comparisons of HTN + comorbid**	Test Statistic	*p-value*
HTN vs. T2DM+HTN	-32.722	1.000
HTN vs. absent	130.918	0.003**
HTN vs. CKD+HTN	-334.002	0.000**
HTN vs. T2DM+CKD+HTN	-486.581	0.005**
T2DM+HTN vs. absent	98.196	1.000
T2DM+HTAE vs. CKD+HTN	-301.280	0.002**
T2DM+HTAE vs. T2DM+CKD+HTN	-453.859	0.031*
absent vs. CKD+HTN	-203.084	0.001**
absent vs. T2DM+CKD+HTN	-355.663	0.118
CKD+HTN vs. T2DM+CKD+HTN	-152.579	1.000

T2DM—type 2 diabetes mellitus; CKD—chronic kidney disease; HTN-Hypertension; p <0.001 **—high significative statistic.

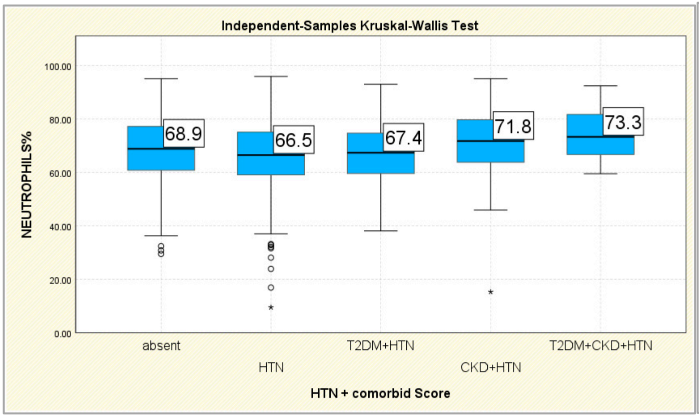

Figure S5. Independent-Sample Kruskal-Wallis Test analysis between diagnostics for neutrophils

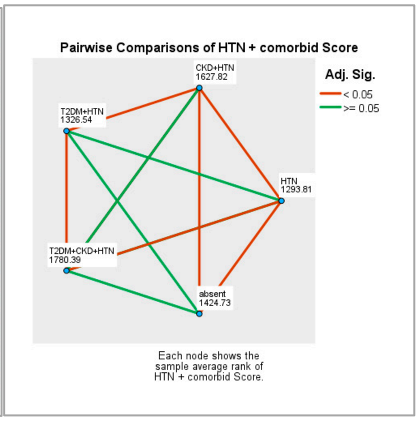

Figure S6. Pairwise Comparisons analysis between diagnostics for neutrophils

Table S12. The Kruskal–Wallis H test-comparative analysis of the abs lute value of lymphocytes on the presence of HTN, with/without CKD or TDM

Lymfocite%	N	Mean	Std. Deviation	Min	Max	Median	IQR 25th	IQR 75th	Kruskal-Wallis H test
**Scor HTN + comorbid**									
absent	714	20.464	10.2645	2.4	64.6	19.600	12.600	27.125	**H = 69.671**
HTN	1537	22.394	9.8149	1.4	80.0	22.100	15.100	28.700	*p <0.001***
T2DM+HTN	142	22.185	9.4312	2.3	48.7	22.400	14.250	28.725	
CKD+HTN	322	18.184	9.0751	2.8	79.3	16.400	11.475	24.500	
TDM+CKD+HTN	33	17.073	7.1198	2.6	29.8	19.000	11.350	21.750	

T2DM—type 2 diabetes mellitus; CKD—chronic kidney disease; HTN-Hypertension; N-number of patients; p <0.001 **—high significative statistic.

Table S13. Pairwise Comparisons analysis between diagnostics for lymphocytes

**Pairwise Comparisons of Scor HTN + comorbid**	Test Statistic	*p-value*
T2DM+CKD+HTN vs. CKD+HTN	73.243	1.000
T2DM+CKD+HTN vs. absent	256.023	0.699
T2DM+CKD+HTN vs. T2DM+HTN	420.655	0.061
T2DM+CKD+HTN vs. HTN	425.534	0.023*
CKD+HTAE vs. absent	182.780	0.006**
CKD+HTAE vs. DZ+HTN	347.412	0.000**
CKD+HTAE vs. HTN	352.291	0.000**
absent vs. T2DM+HTN	-164.632	0.239
absent vs. HTN	-169.511	0.000**

T2DM+HTN vs.	HTN	4.879	1.000

T2DM—type 2 diabetes mellitus; CKD—chronic kidney disease; HTN-Hypertension; p <0.001 **—high significative statistic.

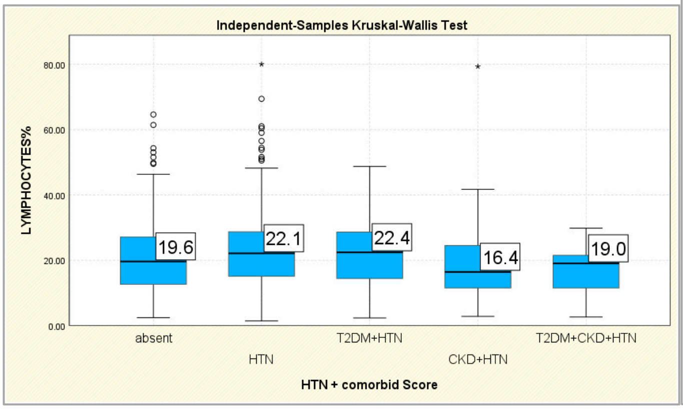

Figure S7. Independent-Sample Kruskal-Wallis Test analysis tics for lymphocytes.

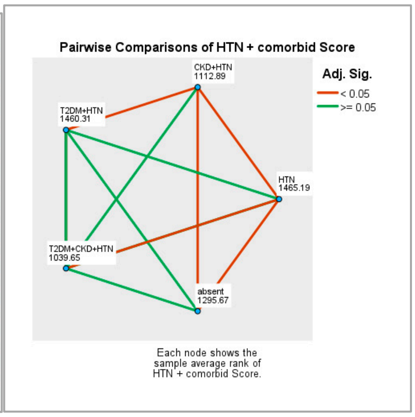

Figure S8. Pairwise Comparisons analysis between diagnos- between diagnostics for lymphocytes

Table S14. The Kruskal–Wallis H test-comparative analysis of the absolute value of platelets on the presence of HTN, with/without CKD or TDM

Platelets	N	Media	Deviația std.	Min	Max	Mediana	IQR 25th	IQR 75th	Kruskal-Wallis H test
**Scor HTN + comorbid**									
absent	714	241.88	105.229	32	798	225.50	176.00	289.00	**H = 13.978**
HTN	1537	247.80	81.283	11	821	243.00	197.00	288.00	*p = 0.007***
T2DM+HTN	142	250.66	87.573	77	600	241.50	197.50	292.25	
CKD+HTN	322	257.44	101.023	62	815	238.00	192.00	299.25	
T2DM+CKD+HTN	33	248.97	92.239	94	444	250.00	170.00	299.00	

T2DM—type 2 diabetes mellitus; CKD—chronic kidney disease; HTN-Hypertension; N-number of patients; p <0.001 **—high significative statistic.

Table S15. Pairwise Comparisons analysis between diagnostics for platelets

**Pairwise Comparisons of Scor HTAE + comorbid**	Test Statistic	*p-value*
absent vs. HTN	-125.138	0.005**
absent vs. T2DM+HTN	-127.425	0.805
absent vs. T2DM+CKD+HTN	-138.971	1.000
absent vs. BRC+HTN	-143.133	0.072
HTAE vs. TDM+HTN	-2.287	1.000
HTAE vs. T2DM+CKD+HTN	-13.834	1.000
HTAE vs. CKD+HTN	-17.995	1.000
T2DM+HTN vs. T2DM+CKD+HTN	-11.546	1.000
T2DM+HTN vs. CKD+HTN	-15.708	1.000
T2DM+CKD+HTN vs. CKD+HTN	4.162	1.000

T2DM—type 2 diabetes mellitus; CKD—chronic kidney disease; HTN-Hypertension; p <0.001

**—high significative statistic.

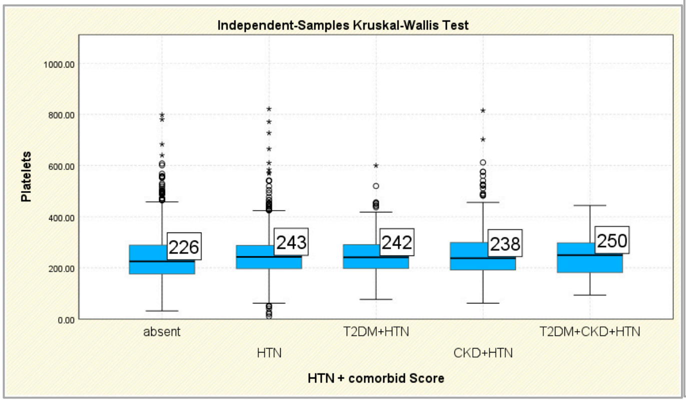

Figure S9. Independent-Sample Kruskal-Wallis Test analysis between diagnostics for platelets

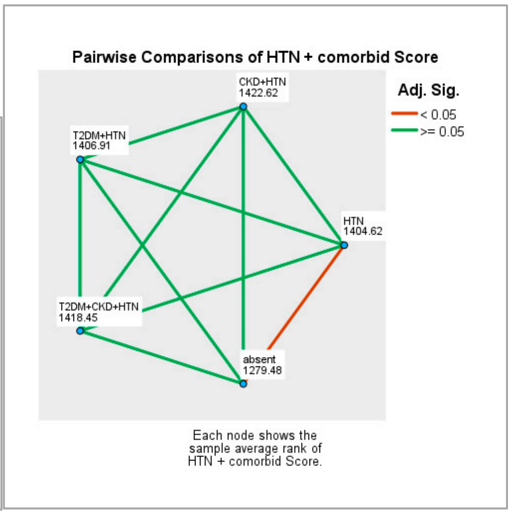

Figure S10. Pairwise Comparisons analysis between diagnostics for platelets

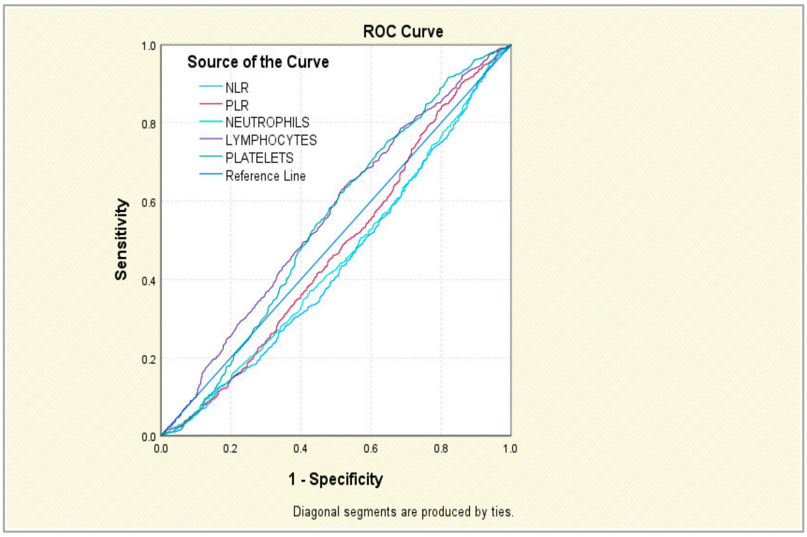

Figure S11. ROC curve of NLR, PLR, neutrophils, lymphocytes, and platelets in HTN .

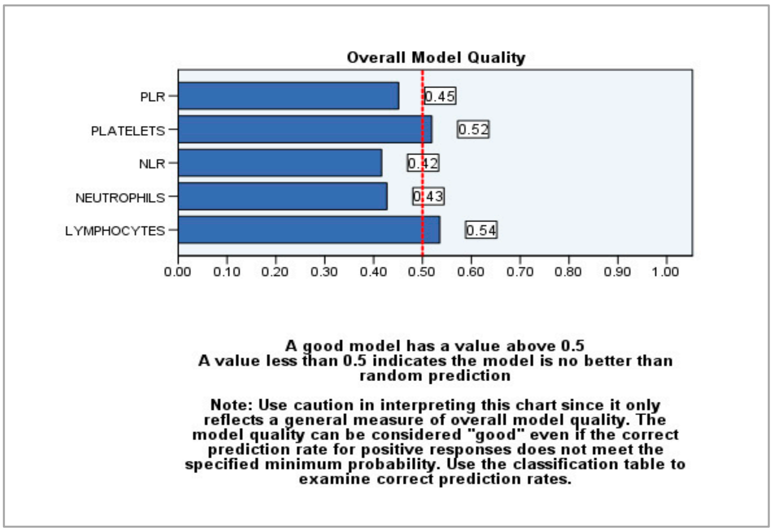

Figure S12. Quality of ROC models of NLR, PLR, neutrophils, lymphocytes, and platelets in HTN. The dashed line represents a non-discriminatory test, a random classifier, which assigns 1 and 0 randomly to the samples for model quality assessment

Table S16. ROC analysis of NLR, PLR, neutrophils, lymphocytes, and platelets in HTN+T2DM diagnoses.

Diagnostic: T2DM + HTN	AUC	*p-value*	95% CI L.inf	95% CI L.sup	Gini Index	Cut-off value	Sensi-tivity	Specific-ity
NLR	0.447	0.038*	0.397	0.497	-0.106	**1.5123**	0.937	0.090
PLR	0.478	0.380	0.429	0.527	-0.044	**5.7069**	0.937	0.130
Neutrophils /µL	0.465	0.171	0.415	0.515	-0.070	**51.750**	0.951	0.081
Lymphocites /µL	0.559	0.021*	0.509	0.610	0.118	**18.950**	0.641	0.485
Platelets /µL	0.546	0.058	0.498	0.595	0.093	**190.50**	0.810	0.314

HTN-Hypertension; N-number of patients; p <0.001 **—high significative statistic

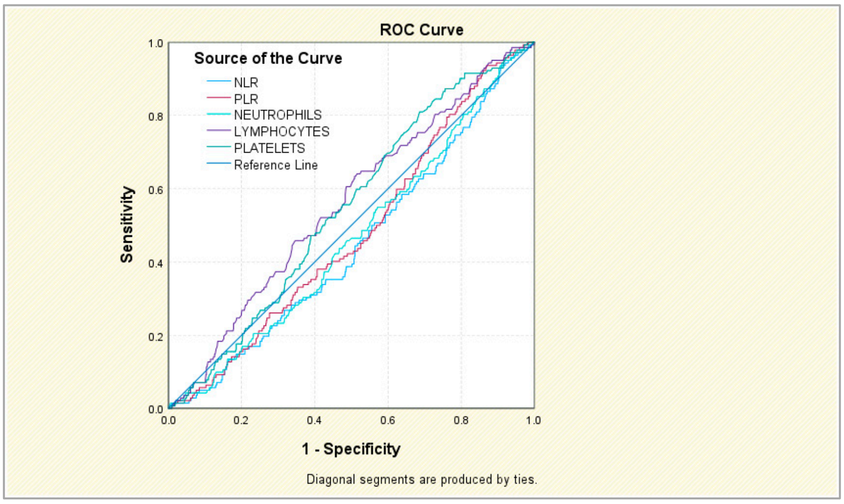

Figure S13. ROC of NLR, PLR, neutrophils, lymphocytes, and platelets in HTN+T2DM

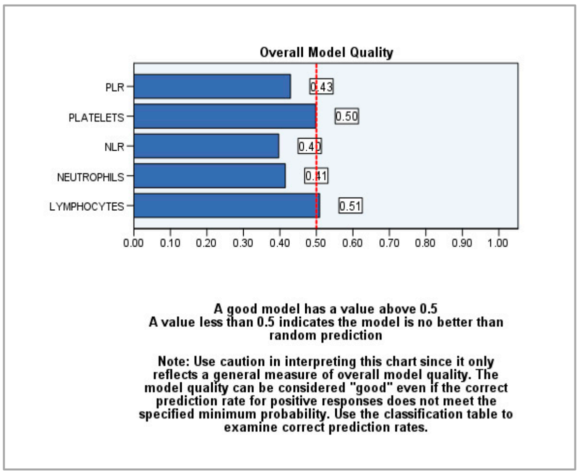

Figure S14. Quality of ROC models of NLR, PLR, neutrophils, lymphocytes, and platelets in HTN+TDM diagnoses.

Table S17. ROC analysis of NLR, PLR, neutrophils, lymphocytes, and platelets in HTN+CKD diagnoses.

**Diagnostic:**			**95% CI**		**Gini**	**Cut-off**	**Sensitiv**	**Specifici**
**CKD + HTN**	**AUC**	***p-value***	L.inf	L.sup	**Index**	**value**	**ity**	**ty**
NLR	0.567	0.000**	0.530	0.603	0.134	**4.4174**	0.506	0.619
PLR	0.580	0.000**	0.543	0.616	0.159	**8.2578**	0.857	0.293
Neutrophils /µL	0.571	0.000**	0.535	0.608	0.143	**73.550**	0.472	0.647
Lymphocites /µL	0.436	0.001**	0.399	0.473	-0.128	**7.850**	0.907	0.112
Platelets /µL	0.551	0.006**	0.515	0.588	0.103	**149.50**	0.922	0.174

HTN-Hypertension; N-number of patients; p <0.001 **—high significative statistic

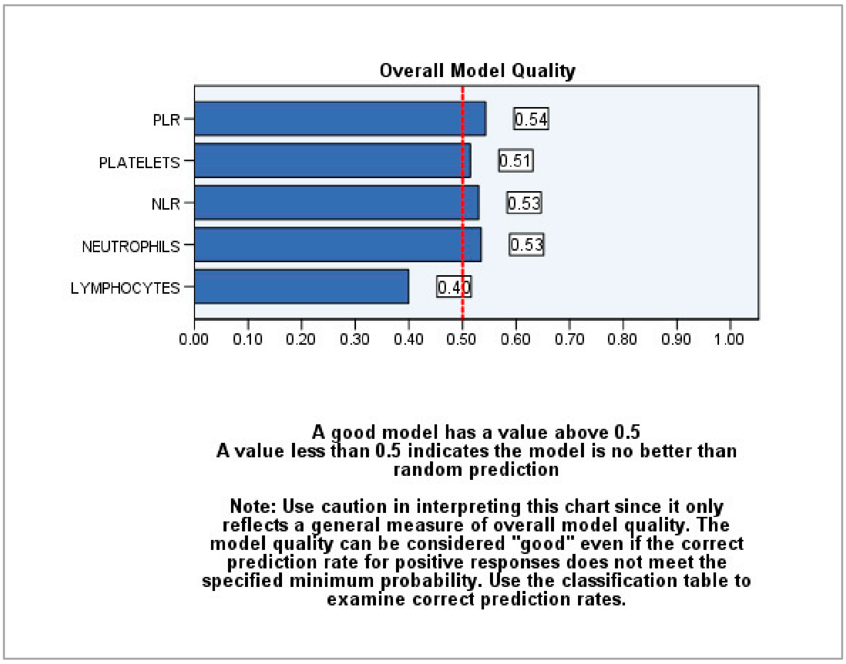

Figure S15. Quality of ROC models of NLR, PLR, neutrophils, lymphocytes, and platelets HTN+CKD diagnoses.

Table S18. ROC analysis of NLR, PLR, neutrophils, lymphocytes, and platelets in HTN+CKD+T2DM diagnoses.

**Diagnostic:**			**95% CI**		**Gini**	**Cut-off**	**Sensitiv**	**Specifici**
**T2DM + CKD + HTN**	**AUC**	*p-value*	L.inf	L.sup	**Index**	**value**	**ity**	**ty**
NLR	0.596	0.020*	0.515	0.677	0.192	**2.6957**	0.879	0.363
PLR	0.589	0.036*	0.506	0.673	0.179	**10.5194**	0.818	0.417
Neutrophils /µL	0.624	0.002**	0.544	0.705	0.249	**59.450**	1.000	0.225
Lymphocites /µL	0.413	0.037*	0.331	0.495	-0.175	**5.000**	0.970	0.042
Platelets /µL	0.544	0.394	0.443	0.645	0.088	**245.50**	0.576	0.598

HTN-Hypertension; N-number of patients; p <0.001 **—high significative statistic

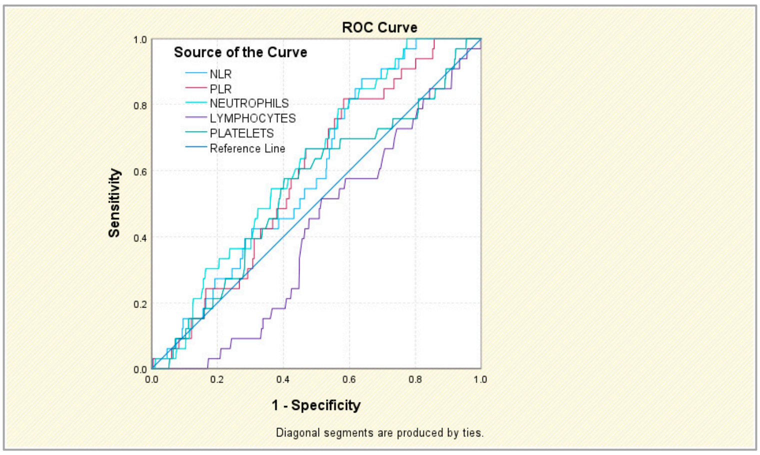

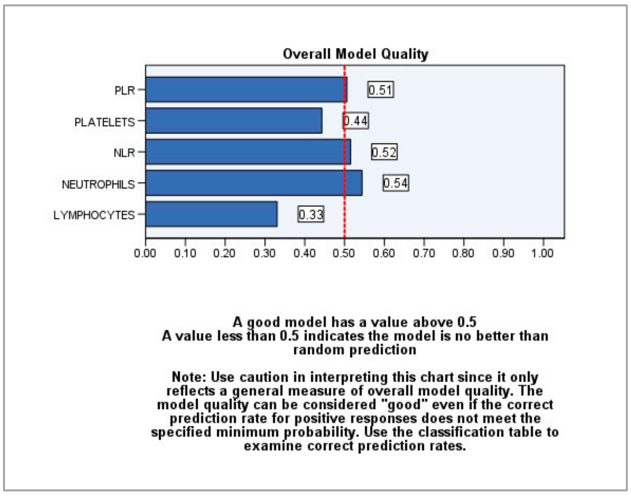

Figure S16. ROC curve of NLR, PLR, neutrophils, lymphocytes**,** lymphocytes, and platelets in HTN+T2DM+CKD diagnoses

Figure S17. Quality of ROC models of NLR, PLR, neutrophils, and platelets in HTN+T2DM+CKD diagnoses
